# Cohort profile: the Korean Youth Health Behavior Panel Survey (KYHPS)

**DOI:** 10.4178/epih.e2026017

**Published:** 2026-04-22

**Authors:** Jun Hyun Hwang, Inho Park, Soon-Woo Park

**Affiliations:** 1 Department of Preventive Medicine, Daegu Catholic University School of Medicine, Daegu, Korea; 2 Department of Statistics & Data Science, Pukyong National University College of Information Technology and Convergence, Busan, Korea

**Keywords:** Adolescent, Health behavior, Cohort studies, Longitudinal studies

## Abstract

The identification of factors that predispose adolescents to adopt healthy lifestyles is essential because this developmental stage shapes long-term health trajectories. To address the lack of longitudinal data on adolescent health in Korea, the Korean Youth Health Behavior Panel Survey (KYHPS) was established in 2019. The KYHPS recruited 5,051 sixth-grade students from 260 elementary schools nationwide, excluding Jeju, and follows them annually for 10 years using a stratified multistage probability sampling design. Data were collected using tablet-assisted self-interviews, and the questionnaires were developed on the basis of the ecological model of health behaviors. The survey focused on 4 major domains—tobacco use, alcohol consumption, diet, and physical activity—along with psychosocial and contextual factors at the individual, family, peer, school, and community levels. Panel retention was 93.1% in Wave 2 and 82.0% in Wave 6. The KYHPS is the first national longitudinal survey specifically focused on adolescent health behaviors in Korea and provides a unique opportunity to examine health trajectories and their determinants across adolescence and into early adulthood, with important implications for prevention and policy.

## INTRODUCTION

Adolescence, defined as the transitional period between childhood and adulthood spanning ages 10 years to 24 years [1], is one of the most rapid and formative stages of human development and is characterized by distinct physical, cognitive, social, emotional, and sexual changes [2]. This period is critical for establishing the foundations of good health and shaping health trajectories and overall well-being across the life course [3,4]. Investment in adolescent health yields a “triple dividend”: it improves health during adolescence, enhances health and well-being in adulthood, and contributes to better outcomes for the next generation [2,3]. Accordingly, the assessment and monitoring of adolescent health behaviors play a pivotal role in identifying risk trajectories, guiding preventive interventions, promoting healthier decision-making, and supporting sustained well-being across the life course [3].

Despite growing recognition of adolescence as a critical period in shaping lifelong health, most health-related data sources for Korean adolescents have been limited to repeated cross-sectional surveys, such as the Korea Youth Risk Behavior Web-based Survey (KYRBS) [5]. The KYRBS is a nationally representative, school-based survey conducted annually among middle and high school students (grades 7–12) across the 17 provinces of Korea using a stratified multistage cluster sampling design. Although such surveys provide valuable prevalence estimates for middle and high school students, they do not permit the tracking of individual trajectories or the identification of predictors of behavioral initiation. As a result, they have limited the ability to identify predisposing factors for health behaviors beyond documenting cross-sectional associations. Moreover, because health behaviors change across school levels [6,7], the existing KYRBS cannot capture transitions during the critical period from elementary to middle school, nor can it track changes as adolescents move into adulthood.

To address this gap, the Korea Disease Control and Prevention Agency (KDCA) and the Ministry of Education established the Korean Youth Health Behavior Panel Survey (KYHPS) in 2019. The KYHPS is a nationally representative longitudinal cohort of sixth-grade students who are followed annually for 10 years, covering the major transitional phase from late childhood through adolescence to early adulthood. It was designed to capture the onset and progression of health behaviors, including alcohol use, tobacco product use, diet, and physical activity, as well as psychosocial factors and contextual influences. By repeatedly assessing the same participants over time, this cohort enables researchers to examine risk trajectories, protective factors, and broader social determinants of adolescent health, thereby providing essential evidence to inform public health interventions and guide national health policy.

## STUDY PARTICIPANTS

### Study population and design

The KYHPS used a multistage cluster sampling design to obtain a nationally representative sample of students enrolled in elementary schools in Korea. The target population comprised sixth-grade students attending elementary schools in Korea in 2019, excluding those in schools located on Jeju Island. Jeju Island was excluded from the sampling frame primarily for efficiency and operational feasibility because of its small population share (approximately 1.45%), which was considered to have limited influence on national estimates. Including a geographically isolated and very small stratum would have substantially increased costs while providing only minimal gains in statistical precision, a concern that is particularly relevant in longitudinal panel surveys. Sixth-grade students were selected for both developmental and methodological reasons. This stage immediately precedes the transition to middle school, allowing the cohort to capture changes in health behaviors across a major educational transition. Including students in lower grades would have required a substantially longer follow-up period before the initiation of key health-risk behaviors could be observed, thereby reducing study efficiency. Furthermore, because the survey was self-administered, sixth-grade students were considered developmentally more capable of providing accurate and reliable responses. The sampling frame was constructed from the official nationwide school administrative database provided by the Korean Educational Development Institute on the basis of records as of April 2018. This database includes all registered elementary schools nationwide and therefore provides near-complete coverage of the eligible target population. The sampling process consisted of 3 stages: stratification, allocation, and selection. During stratification, 25 strata were constructed according to province and region size (metropolitan, small- and medium-sized cities, and rural areas). A compromise allocation method was used to secure an adequate number of students not only at the national level but also within each province and region size category. Specifically, the number of schools in each province was allocated proportionally to the square root of the number of schools, whereas within each province, the number of schools by region size was allocated in proportion to the number of schools. At least 2 schools were assigned to each stratum. A stratified multistage probability sampling method was then applied, with schools as the primary sampling units and classes as the secondary sampling units. Within each stratum, the school list was ordered by province, district, and school size, measured by the number of sixth-grade classes. Schools were subsequently selected by systematic probability sampling with probability proportional to size. Because the unit of selection within schools was the class rather than the individual student, the number of classes was considered a more appropriate measure of size for achieving stable self-weighting. A total of 260 schools were selected from a sampling frame of 3,741 eligible elementary schools using this stratified multistage probability sampling design. Within each sampled school, 2 or 3 classes were randomly selected, and all students in the selected classes were included in the survey sample [8].

### Enrollment and follow-up

#### Enrollment

The KYHPS panel was established with 5,051 sixth-grade students from 260 elementary schools nationwide, together with 1 parent or guardian for each student and 1 representative from each participating school, typically a health teacher. Selected students are followed annually for 10 years, from the sixth grade of elementary school to 3 years after high school graduation. Parents or guardians participate in the survey through Wave 7, corresponding to the third year of high school, when students reach the age of majority. School representatives participated only in the baseline survey, which was completed in 257 schools because 3 of the 260 sampled schools declined to participate in the school survey [8].

#### Baseline characteristics

At baseline, the weighted sample consisted of 51.5% boys and 48.5% girls. By residential area, the largest proportion of students lived in Gyeonggi-do (28.1%), followed by Seoul (15.9%), whereas smaller proportions lived in Sejong (0.9%) and Ulsan (2.9%). Most students rated their subjective academic performance as middle (40.2%) or upper-middle (38.9%), and only 0.9% rated it as low. Similarly, most students perceived their household economic status as middle (56.4%) or upper-middle (26.7%), whereas 0.6% reported it as low (Table 1). In addition to demographic characteristics, several key health behavior indicators were included to provide a more comprehensive baseline profile of the panel participants, including lifetime tobacco product use, lifetime alcohol use (sip- and glass-based), skipping breakfast on at least 5 days per week, fast-food consumption at least 3 times per week, and engaging in at least 60 minutes of physical activity on at least 5 days per week, as shown in Table 1.

#### Retention

**Figure 1.**
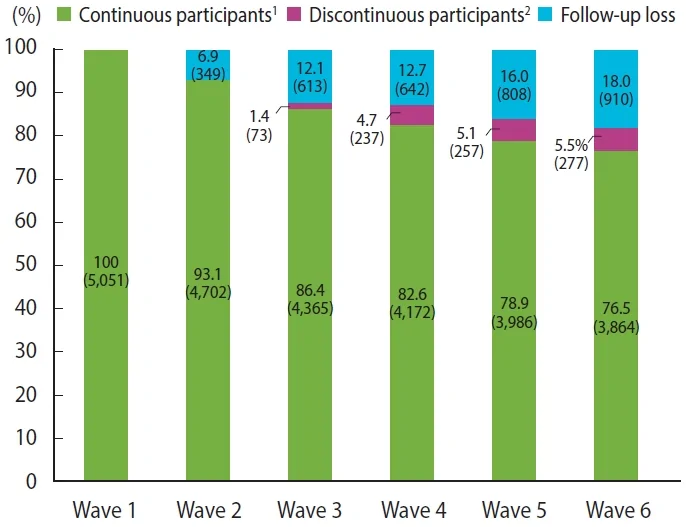
Panel retention rates of the Korean Youth Health Behavior Panel Survey from Wave 1 (baseline, 2019) to Wave 6 (2024). Values are presented as % (number). 1Continuous participants: participated in all waves up to the given year. 2Discontinuous participants: missed at least 1 previous wave but participated in the given wave.

Among the 5,051 sixth-grade students from 260 elementary schools who participated in the baseline survey, discontinuous participation, defined as missing 1 or 2 survey waves but returning in later waves and being counted under cross-sectional weights, was first observed in Wave 3. When these participants were included, panel retention rates were 93.1% (n=4,702 students) in Wave 2, 87.8% (n=4,438) in Wave 3, 87.3% (n=4,409) in Wave 4, 84.0% (n=4,243) in Wave 5, and 82.0% (n=4,141) in Wave 6. When only continuous participants, defined as those who completed every survey wave up to the given year and were used for longitudinal weight estimation, were considered, panel retention rates were slightly lower: 86.4% (n=4,365) in Wave 3, 82.6% (n=4,172) in Wave 4, 78.9% (n=3,986) in Wave 5, and 76.5% (n=3,864) in Wave 6 (Figure 1) [8].

To assess potential selection bias due to attrition, baseline characteristics were compared between participants who did and did not participate in Wave 6. The participation rate was significantly higher among boys than among girls (83.3 vs. 80.5%, p=0.009) and significantly lower among students from households reporting the highest economic status (77.8%, p=0.002). The relatively lower participation rate in the highest-economic-status group may indicate that survey incentives were less likely to motivate continued participation in this group. However, no significant differences were observed with respect to baseline academic performance, lifetime tobacco product use, lifetime alcohol use, skipping breakfast on at least 5 days per week, fast-food consumption at least 3 times per week, or engaging in 60 or more minutes of physical activity on at least 5 days per week (Table 2).

To minimize attrition, data collection was conducted by a nationwide professional survey agency with extensive experience in longitudinal panel studies. The same trained interviewers followed participants across waves to foster rapport and trust. Survey timing and locations were arranged flexibly to accommodate participants’ schedules. Modest incentives were provided to encourage continued participation. In addition, participants were contacted on major occasions, such as national holidays or birthdays, to maintain engagement with the panel.

For analyses examining change over time, longitudinal weights restricted to respondents who participated in all relevant waves should be applied. Cross-sectional weights should be used when analyzing outcomes at a specific time point.

### Ethics statement

The study protocol was approved by the Institutional Review Board of Daegu Catholic University Medical Center (CR-19-107).

## MEASUREMENTS

### Survey items

Adolescence is the life stage during which exposure to the social determinants of health is most dynamic and diverse [9]. During this period, influences beyond the family environment become increasingly important, with peers, the media, and education playing major roles. At the same time, community-level and structural-level determinants continue to shape adolescent health trajectories [3]. In line with this perspective, the KYHPS survey instrument was developed on the basis of the ecological model of health behaviors. Initially, the KYHPS focused on 4 key domains of adolescent health behaviors: tobacco use, which was the primary focus, alcohol consumption, diet, and physical activity. Within each domain, both key behavioral variables and their predisposing factors were assessed. Consistent with the ecological model, questionnaire items were organized according to levels of influence, including individual, family, peer, school, and community factors.

The questionnaire was developed on the basis of results from a preliminary study conducted from 2017 to 2018 to establish the KYHPS. This preliminary study was informed by a nationwide literature review, including the KYRBS and international surveys targeting adolescents, and the questionnaire items were developed through comprehension and reliability testing [10]. For items introduced in later waves, outcome-related variables were primarily adopted from existing national surveys to ensure comparability and established validity. For other newly added items, particularly those assessing additional determinants or contextual factors, comprehension testing and pilot testing were conducted before implementation.

Table 3 provides an overview of the major survey domains and representative questionnaire items administered across survey waves. The tobacco use domain, a major focus of the KYHPS, included use of various tobacco products as well as items not covered in the KYRBS, such as experience with flavored tobacco. In Waves 1–4, an introductory screening question assessed lifetime use of any tobacco product regardless of product type. Beginning in Wave 5, this item was removed, and respondents were asked directly about their experience with individual tobacco products. The domain also included questions on perceptions, attitudes, and norms related to tobacco use. In Wave 5, additional items were introduced to assess perceptions of the relative harmfulness of e-cigarettes and heated tobacco products, as well as exposure to advertising and promotional activities and attitudes toward these products. The alcohol use domain included items on sipping-based drinking, which have not been included in previous national surveys in Korea, to evaluate the significance of early sipping experiences in shaping adolescent drinking behaviors [11] within the Korean context. Subsequently, items assessing behavioral changes related to the coronavirus disease 2019 (COVID-19) pandemic were added in Waves 3–4 (2021–2022). In Wave 3 (2021), the Generalized Anxiety Disorder 7-item scale and the Family Affluence Scale were incorporated. Wave 5 (2023) included additional items on subjective health status and physician-diagnosed conditions, including asthma and allergic rhinitis (Table 3).

The parent questionnaire, which consisted of 33 items in Wave 1, addressed family relationships with the student as well as family-level factors that may influence the development of the student’s health behaviors. The school questionnaire, administered only in Wave 1, was designed to assess the level of interest in student health and related activities at the elementary school level. Detailed questionnaire items, including those administered to parents and school representatives, are available in the official KYHPS user guide and statistical reports [8,12].

### Survey method

From Waves 1 to 5, students completed the survey using tablet-assisted self-interviews (TASI) after receiving instructions from trained interviewers who visited their households. This protocol was maintained during the COVID-19 pandemic, with interviewers continuing to conduct individual household visits. The parent survey was administered using the same method as the student survey through Wave 3; beginning in Wave 4, telephone interviews were also permitted. School representatives completed an online self-administered questionnaire.

In Wave 1, after sample schools had been selected and preparatory procedures completed, the survey was conducted from September 2019 to early January 2020. In Waves 2 and 3 (2020–2021), the survey period was delayed by approximately 2 months from the originally scheduled start in May because of the COVID-19 pandemic and was instead conducted between July and December. Beginning in Wave 4 (2022), the survey schedule returned to normal, with data collection taking place from May to September. The Wave 5 survey (2023) was conducted from May to November, and the Wave 6 survey (2024) from April to October, with most students participating during the first semester.

## KEY FINDINGS

Since Wave 2, the KYHPS has published annual statistical reports presenting the prevalence and incidence of major health behaviors.

### Tobacco product use

Annual incidence rates of lifetime tobacco product use by product type, including conventional cigarettes, e-cigarettes, and heated tobacco products, were calculated for each grade. Among participants who were continuously followed through Wave 6, the annual incidence of tobacco product initiation increased with grade progression: 0.29% in the first year of middle school, 1.34% in the second year of middle school, 2.38% in the third year of middle school, 3.29% upon entry into high school, and 3.22% in the second year of high school. In addition, longitudinal follow-up data showed that among adolescents who reported experience with 2 or more tobacco products, the first product used was most commonly conventional cigarettes (60.7%), followed by e-cigarettes (36.3%), heated tobacco products (1.8%), and unknown products (1.2%) [8].

Notably, in Wave 6 (11th grade), the prevalence of current e-cigarette use among female students exceeded that of conventional cigarette use for the first time [8]. This pattern has not yet been observed in the KYRBS [7], underscoring the need for continued surveillance. In Wave 6, 72.6% of current e-cigarette users were dual or multiple users who also used conventional cigarettes or heated tobacco products. This proportion was higher than that observed among current cigarette smokers (64.9%). In addition, 50.2% of e-cigarette users reported having attempted to quit, compared with 75.1% of cigarette smokers. Furthermore, 77.3% of adolescents with lifetime tobacco product use reported initiating use with a flavored product [8,13].

### Alcohol use

Annual incidence rates of alcohol initiation also increased across grade levels. An analysis of data from Waves 1–5 identified several significant predisposing factors for initiation, including male sex, prior experience with sipping alcohol, intention to drink in the future, low perceived risk associated with alcohol use, having family members who drink, exposure to drinking scenes in the media, and witnessing alcohol consumption in public spaces [14]. The adjusted odds ratio for alcohol initiation among participants with prior sipping experience was 2.83 (95% confidence interval, 2.39 to 3.36) [13].

## STRENGTHS AND WEAKNESSES

The KYHPS has several strengths. First, to our knowledge, it is the first national longitudinal survey specifically designed to examine health behaviors among Korean adolescents. Although other longitudinal studies of young people, such as the Korean Children and Youth Panel Survey and the Korean Education Longitudinal Study, have been conducted, these surveys have focused primarily on education or psychosocial development. The KYHPS is the first to have health as its central focus. Second, the KYHPS follows adolescents from the sixth grade of elementary school through early adulthood, allowing observation of critical transitional periods during which health behaviors are likely to change. Third, during the COVID-19 pandemic, the survey was consistently conducted through household visits using the same TASI method, thereby reducing the potential for differences attributable to survey mode effects.

However, several limitations should be noted. First, the KYHPS has limitations in representativeness. Jeju Province was excluded from the sampling frame, and selection bias may also have arisen from the relatively low within-class participation rate (5,051 participants out of 18,411 students contacted across 2–3 classes per sampled school; 27.4%) [15] compared with that of the KYRBS (95.3% in 2019) [16]. Second, because the KYHPS includes sensitive questions on tobacco and alcohol use, responses may be subject to social desirability bias [17,18]. Given the longitudinal design, underreporting may be more likely than in anonymous cross-sectional surveys. For these 2 reasons, the number of adolescents reporting smoking or drinking experiences during follow-up was relatively small; therefore, caution is warranted when conducting related analyses or interpreting the findings. Third, all data, including height and weight, were collected through self-reported questionnaires rather than through clinical verification, such as physical measurements or cotinine testing. As a result, logical inconsistencies may arise in items such as lifetime tobacco or alcohol use, for example, when lifetime use is reported in 1 year but not in the following year. However, to minimize potential measurement bias, strict confidentiality of responses was ensured, and the survey was self-administered in a private setting. In addition, when logical inconsistencies were detected, a pop-up notification prompted participants to review and re-enter their responses on the basis of their previous answers.

The KYHPS offers a unique opportunity to examine how family, peers, and the broader social environment shape adolescent health behaviors over time. This survey is expected to provide essential evidence for understanding patterns of change in adolescent health behaviors and to serve as a foundation for the development of effective adolescent health policies.

## Figures and Tables

**Table 1.** Baseline demographic characteristics of participants in the Korean Youth Health Behavior Panel Survey (Wave 1)

Classification	n	Unweighted %	Weighted %
Sex			
Male	2,634	52.1	51.5
Female	2,417	47.9	48.5
Residential area			
Seoul	516	10.2	15.9
Busan	359	7.1	5.7
Daegu	301	6.0	4.9
Incheon	368	7.3	5.9
Gwangju	284	5.6	3.4
Daejeon	212	4.2	3.1
Ulsan	221	4.4	2.9
Sejong	90	1.8	0.9
Gyeonggi-do	801	15.9	28.1
Gangwon-do	210	4.2	2.9
Chungcheongbuk-do	346	6.9	3.2
Chungcheongnam-do	235	4.7	4.5
Jeollabuk-do	273	5.4	3.7
Jeollanam-do	261	5.2	3.5
Gyeongsangbuk-do	296	5.9	4.6
Gyeongsangnam-do	278	5.5	6.7
Subjective academic performance			
High	687	13.6	14.3
Upper-middle	1,944	38.5	38.9
Middle	2,053	40.7	40.2
Lower-middle	319	6.3	5.8
Low	48	1.0	0.9
Subjective economic status			
High	573	11.3	10.9
Upper-middle	1,340	26.5	26.7
Middle	2,851	56.4	56.4
Lower-middle	256	5.1	5.4
Low	31	0.6	0.6
Health behaviors			
Lifetime tobacco product use	16	0.3	0.4
Lifetime alcohol use (sip-based)	1,852	36.7	36.4
Lifetime alcohol use (drink-based)	408	8.1	7.5
Skipping breakfast ≥5 day/wk	929	18.4	17.9
Fast food consumption ≥3 times/wk	1,061	21.0	20.9
Physical activity for at least 60 min ≥5 times/wk	1,516	30.0	29.8

**Table 2.** Comparison of baseline characteristics between participants and non-participants in Wave 6 of the Korean Youth Health Behavior Panel Survey

Classification	n	Unweighted %		p-value
		Participants (n=4,141)^1^	Non-participants (n=910)	
Sex				0.009
Male	2,634	83.3	16.7	
Female	2,417	80.5	19.5	
Subjective academic performance				0.141
High	687	83.7	16.3	
Upper-middle	1,944	82.0	18.0	
Middle	2,053	82.2	17.8	
Lower-middle	319	78.4	21.6	
Low	48	72.9	27.1	
Subjective economic status				0.002
High	573	77.8	22.2	
Upper-middle	1,340	80.2	19.8	
Middle	2,851	83.2	16.8	
Lower-middle	256	86.3	13.7	
Low	31	90.3	9.7	
Health behaviors				
Lifetime tobacco product use	16	0.3	0.6	0.187
Lifetime alcohol use (sip-based)	1,852	36.9	35.5	0.418
Lifetime alcohol use (drink-based)	408	8.0	8.4	0.738
Skipping breakfast ≥5 day/wk	929	18.0	20.3	0.096
Fast food consumption ≥3 times/wk	1,061	21.1	20.8	0.847
Physical activity for at least 60 min ≥5 times/wk	1,516	30.3	28.6	0.294

1 Included discontinuous participants (n=277).

**Table 3.** Overview of student survey items across waves in the Korean Youth Health Behavior Panel Survey (Wave 1–6)

Survey domains	1st (2019)	2nd (2020)	3rd (2021)	4th (2022)	5th (2023)	6th (2024)
	Sep 2019–Jan 2020	Jul–Dec 2020	Jul– Dec 2021	May–Sep 2022	May–Nov 2023	Apr–Oct 2024
Tobacco use						
Lifetime tobacco product use	X	X	X	X	-	-
Lifetime cigarette/e-cigarette/heated tobacco product use	X	X	X	X	X	X
Past 30 days cigarette/e-cigarette/heated tobacco product use	X	X	X	X	X	X
Age at tobacco initiation	X	X	X	X	X	X
Reason for initiation	X	X	X	X	X	X
Quit attempts and quit intention	X	X	X	X	X	X
First type of tobacco product used	X	X	X	X	X	X
Flavored tobacco product use (at initiation/past 30 days)	X	X	X	X	X	X
Future intention to use tobacco products	X	X	X	X	X	X
Relative harmfulness of e-cigarettes and heated tobacco products	-	-	-	-	X	X
Exposure to advertising and promotion for e-cigarettes and heated tobacco products	-	-	-	-	X	X
Attitudes toward e-cigarettes and heated tobacco products	-	-	-	-	X	X
Alcohol use						
Lifetime alcohol use (sip-based)	X	X	X	X	X	X
Lifetime alcohol use (drink-based)	X	X	X	X	X	X
Age at drinking initiation	X	X	X	X	X	X
Reason for initiation	X	X	X	X	X	X
Past 30 days drinking frequency	X	X	X	X	X	X
Average drinking amount	X	X	X	X	X	X
Future drinking intention	X	X	X	X	X	X
Dietary behaviors						
Breakfast skipping	X	X	X	X	X	X
Fruit intake	X	X	X	X	X	X
Vegetable intake	X	X	X	X	X	X
Fast food consumption	X	X	X	X	X	X
Sugar-sweetened beverage consumption	X	X	X	X	X	X
Physical activity						
Physical activity (≥60 min) per day	X	X	X	X	X	X
Vigorous physical activity	X	X	X	X	X	X
Muscle strengthening exercise	X	X	X	X	X	X
Walking duration	X	X	X	X	X	X
Sedentary time	X	X	X	X	X	X
Psychosocial factors and others						
Perceived stress	X	X	X	X	X	X
Depressive symptoms	X	X	X	X	X	X
Smartphone use time	X	X	X	X	X	X
Smartphone dependency	-	-	-	-	-	X
Sleep duration	X	X	X	X	X	X
Subjective academic achievement	X	X	X	X	X	X
Subjective economic status	X	X	X	X	X	X
Self-reported height and weight	X	X	X	X	X	X
Additional module						
COVID-19 related behavioral changes	-	-	X	X	-	-
GAD-7	-	-	X	X	X	X
FAS	-	-	X	X	X	X
Subjective health status	-	-	-	-	X	X
Physician-diagnosed asthma	-	-	-	-	X	X
Physician-diagnosed allergic rhinitis	-	-	-	-	X	X

COVID-19, coronavirus disease 2019; GAD-7, Generalized Anxiety Disorder 7-item; FAS, Family Affluence Scale.

## Data Availability

Raw KYHPS data will be made publicly available in October 2025 through the KDCA website and the Microdata Integrated Service of Statistics Korea (http://mdis.kostat.go.kr). Researchers interested in using the raw data for research should contact the Division of Climate Change and Health Hazard, Department of Health Hazard Response, KDCA (e-mail: kdcakyhps@korea.kr).
